# Insights into the Genetic Profile of Two Siblings Affected by Unverricht-Lundborg Disease Using Patient-Derived hiPSCs

**DOI:** 10.3390/cells11213491

**Published:** 2022-11-04

**Authors:** Valeria Lucchino, Luana Scaramuzzino, Stefania Scalise, Michela Lo Conte, Clara Zannino, Giorgia Lucia Benedetto, Umberto Aguglia, Edoardo Ferlazzo, Giovanni Cuda, Elvira Immacolata Parrotta

**Affiliations:** 1 Department of Experimental and Clinical Medicine, University Magna Graecia, 88100 Catanzaro, Italy; 2 Department of Medical and Surgical Sciences, University Magna Graecia, 88100 Catanzaro, Italy

**Keywords:** Unverricht-Lundborg disease, EPM1, cystatin B, human induced pluripotent stem cells, iPSCs-derived neurons, disease model

## Abstract

Unverricht-Lundborg disease (ULD), also known as progressive myoclonic epilepsy 1 (EPM1), is a rare autosomal recessive neurodegenerative disorder characterized by a complex symptomatology that includes action- and stimulus-sensitive myoclonus and tonic-clonic seizures. The main cause of the onset and development of ULD is a repeat expansion of a dodecamer sequence localized in the promoter region of the gene encoding cystatin B (CSTB), an inhibitor of lysosomal proteases. Although this is the predominant mutation found in most patients, the physio-pathological mechanisms underlying the disease complexity remain largely unknown. In this work, we used patient-specific iPSCs and their neuronal derivatives to gain insight into the molecular and genetic machinery responsible for the disease in two Italian siblings affected by different phenotypes of ULD. Specifically, fragment length analysis on amplified CSTB promoters found homozygous status for dodecamer expansion in both patients and showed that the number of dodecamer repeats is the same in both. Furthermore, the luciferase reporter assay showed that the CSTB promoter activity was similarly reduced in both lines compared to the control. This information allowed us to draw important conclusions: (1) the phenotypic differences of the patients do not seem to be strictly dependent on the genetic mutation around the CSTB gene, and (2) that some other molecular mechanisms, not yet clearly identified, might be taken into account. In line with the inhibitory role of cystatin B on cathepsins, molecular investigations performed on iPSCs-derived neurons showed an increased expression of lysosomal cathepsins (B, D, and L) and a reduced expression of CSTB protein. Intriguingly, the increase in cathepsin expression does not appear to be correlated with the residual amount of CSTB, suggesting that other mechanisms, in addition to the regulation of cathepsins, could be involved in the pathological complexity of the disease.

## 1. Introduction

Unverricht-Lundborg disease (ULD) is the most common form of progressive myoclonic epilepsy (PME), a group of rare neurodegenerative diseases with debilitating progression [1,2]. ULD is an autosomal recessively inherited disorder that affects young people between the ages of six and 16 years and it is known to occur worldwide, with a greater incidence in Finland (1:20,000 births per year), but also in some Mediterranean countries (i.e., Italy, France, Tunisia, Algeria, and Morocco) [3,4]. To date, ULD is not fatal, but it is very serious due to poor prognosis and resistance to treatments—antiepileptic drugs such as valproate and benzodiazepines are only symptomatic and supportive [5]. The clinical symptoms are myoclonus (involuntary contraction of the muscles), tonic-clonic epileptic seizures, intention tremor, cerebellar ataxia, photosensitivity, dysarthria and mild mental deterioration [6]. As the disease progresses, myoclonic and epileptic events tend to occur more intensely and frequently, increasing patients’ disability. The primary genetic causes of ULD are mutations in the gene encoding cystatin B (CSTB), an endogenous inhibitor of cysteine proteases such as lysosomal cathepsins. Specifically, the most common mutation described in patients affected by ULD is an unstable expansion (>30 times) of a dodecamer repeat sequence (5′-CCCCGCCCCGCCG-3′) located in the 5′-UTR of the CSTB gene promoter, 175 bp upstream of the transcription start codon [7,8,9,10,11,12]. In healthy subjects, the dodecamer repeat is present in two to three copies, while affected individuals contain large expansions (30 to 80) of the dodecamer repeat [9,10]. This expansion mutation is mostly detected in homozygous form, but it can also occur in a compound heterozygous form together with CSTB coding-region point mutations [12]. No correlation between repeat size and age at onset or severity of the disease has been reported [6,13]. The aberrant expansion of the dodecamer repeat sequence is causative of downregulation of CSTB gene and protein expression and, consequently, of its inhibitory activity [14]. Although the genetic causes of Unverricht-Lundborg disease are known, the molecular mechanisms underlying the disease have not yet been fully elucidated. At the cellular level, cystatin B was found to be mainly localised in the nucleus of proliferating cells, while in differentiated cells it can be also found in cytoplasm [15] and lysosomes [16]. The distribution of CSTB in many subcellular compartments reflects the fact that it could perform different functions beyond inhibition of proteases.

**Figure 1:**
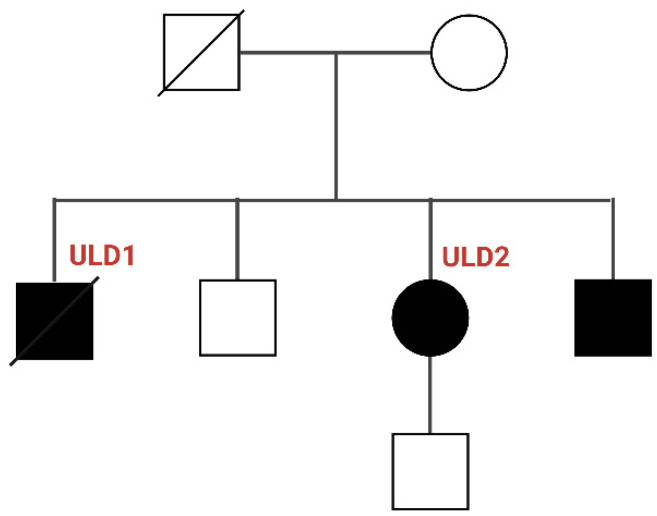
Family pedigree of ULD patients studied (created with Biorender). *Open symbols*: unaffected individuals, *solid symbols*: affected individuals, *squares*: males, *circles*: females, *symbols with a diagonal line*: deceased.

It was previously reported that in the brain Cystatin B is involved in many biological processes. Particularly, it protects cerebral cells against apoptotic processes by regulating cathepsin’s proteolytic activity [17]. Analyses of the brains of mice knocked out of CSTB, which display many of the clinical features of human ULD [18], revealed the presence of apoptosis associated with gliosis, leading to a marked loss of granule cells and Purkinje cells in the cerebellar granular layer [17]. Furthermore, Cystatin B has also been shown to be involved in synaptic plasticity and regulation of reduction-oxidative homeostasis [19], it affects neuronal progenitors’ proliferation, interneurons migration and regulates cell cycle progression [20]. Microarray-based gene expression analyses on neurons and cerebellar tissue of CSTB^−/−^ mice showed expression changes in synaptogenesis and immune response genes, as well as alterations in GABAergic signalling [17]. It has been hypothesised that the loss of CSTB inhibition of lysosomal proteases triggers apoptosis of GABAergic inhibitory neurons, with a consequent increase of neuronal excitability; the imbalance of excitatory/inhibitory neurons could be responsible of epileptic seizures and myoclonic events [12,14,21,22]. Finally, in 2002, Di Giaimo and collaborators identified three brain-specific non-proteases that seem to interact with cystatin B in vivo: rat neurofilament light peptide (RACK-1), rat brain β-spectrin and Neurofilament (NF-L) [23], further confirming that CSTB may also have other, still unidentified, molecular function than solely cathepsin inhibition. In this study, we generated induced pluripotent stem cells (iPSCs) from two Italian siblings (ULD1 and ULD2) belonging to a clinically well characterised Mediterranean myoclonus family [3] (Figure 1).

The two siblings analyzed in this study are affected by different phenotypic and clinical degrees of ULD: ULD1 was affected by a severe form of the disease, while ULD2 has a very mild form of ULD. The age of onset of the disease was 11 years for ULD1 and 12 years for ULD2, and tonic-clonic seizures were the first sign of the disease for both patients (Table 1).

Patient-specific induced pluripotent stem cells are a powerful tool for the in vitro study of diseased cell types that are otherwise inaccessible, such as cardiomyocytes and neurons [24,25], as they retain the genetic and epigenetic information of the patient’s somatic cells from which they are derived and, like human embryonic stem cells (hESCs), they have the potential to differentiate into all specialized cell types [26]. Even though it was reported that iPSCs have some molecular and biochemical differences compared to hESCs [27], they offer many advantages over hESCs, as their use in research and clinics overcomes ethical and immune rejection issues [28]. Indeed, iPSCs are currently used in many biochemical applications, such as basic research, cell therapy, drug screening, and human disease modelling [29]. On this basis, taking advantage of iPSCs technology, here we characterize the genetic mutation of the two patients with ULD and measure the expression levels of cystatin B and cathepsins in neurons derived from patients with ULD. Interestingly, our results show that the two siblings have the same mutation in the *CSTB* gene promoter, although they are affected by different clinical phenotypes. As expected, due to the homozygosity of the dodecamer expansion mutation, the *CSTB* promoter is less active in ULD1 and ULD2 compared to the control, and this results in a significantly reduced expression of cystatin B in patient-derived neurons. Analysis of the expression levels of cathepsins in ULD patient’s neurons revealed a tendency of increase, not proportional to the residual amount of *CSTB*, suggesting that other mechanism(s) apart from the regulation of cathepsins by cystatin B, could be involved and therefore responsible for the different degrees of disease severity. In this regard, we investigated the ability of activated cathepsins to trigger apoptotic cell death. There is evidence that cathepsins, particularly B and D, are involved in caspase-independent PARP-1 cleavage, which is a marker of apoptosis [30]. As a result, we found that ULD patients express comparable, significantly higher expression of cleaved PARP1 compared to control, suggesting that improperly activated cathepsins could initiate programmed cell death via PARP1 cleavage.

## 2. Materials and Methods

### 2.1. Generation of Human Induced Pluripotent Stem Cells (hiPSCs) from Two Siblings Affected by ULD

In this study, we generated iPSCs from two Italian siblings affected by two different clinical phenotypes of ULD. The detailed description of iPSCs generation from ULD1 and ULD2 (lines *UNIMGi003-A* and *UNIMGi004-A*, respectively) is described in [31]. The generation of iPSCs from the healthy subject used as control in all our experiments is described in [32] (see hiPSCs-3 line). Briefly, we generated control iPS cell line through genetic reprogramming of skin fibroblasts of a healthy Caucasian male, having the same age of our ULD patients. iPSCs were cultured on Matrigel-coated (Corning, Corning, NY, USA) plates and maintained in mTeSR™ Plus medium (Stem Cell Technologies, Vancouver, BC, Canada) in a humidified incubator at 37 °C at 5% CO_2_. Medium was replaced every other day and cells were passaged every 4 days as clumps using Gentle Dissociation reagent (Stem Cell Technologies, Vancouver, BC, Canada). All cell lines were regularly tested for Mycoplasma before being used in experiments with the Mycoplasma PCR Detection Kit (Applied Biological Materials, Richmond, BC, Canada).

### 2.2. Generation of Neurons from Human Induced Pluripotent Stem Cells

iPSC-derived neurons were obtained as previously described [33], with some modifications. Briefly, control and ULD-hiPSCs were induced toward neural stem cells (NSCs) differentiation using Gibco^®^ PSC Neural Induction Medium (Thermofisher Scientific, Waltham, MA, USA), according to manufacturer’s instructions. For the neuronal differentiation, NSCs were seeded at the density of 5 × 10^6^ on 10-mm dishes pre-treated with 1× Poly-D-lysine hydrobromide having a molecular weight of 30,000–70,000 (Merck, Darmstadt, Germany) and 5 μg/mL Laminin (Sigma-Aldrich, St. Louis, MO, USA). Neurons were cultured in Neuronal Differentiation Medium (NDMC) composed of Neurobasal Medium, 1× B27™, 1× GlutaMAX™, 2× CultureOne™ Supplement, 200 μM L-ascorbic acid and 0.2% Penicillin/Streptomycin (all from Thermo Fisher Scientific, Waltham, MA, USA). NDMC was completed with GDNF (10 ng/mL) and BDNF (20 ng/mL) (both from PeproTech, London, UK). After 6 days, cytokine concentration was reduced to 5 ng/mL for GDNF and to 10 ng/mL for BDNF. On day 15 of differentiation, GDNF was removed while BDNF was kept at 10 ng/mL. Neurons were maintained for 21 days in a humidified incubator at 37 °C at 5% CO_2_ and differentiation medium refreshed every three days. At day 21 of differentiation, neurons were harvested and processed for characterization (Figure S3) and for molecular validation of genetic data.

### 2.3. DNA Extraction

Genomic DNA was extracted from CTRL and ULD-hiPSCs as follows: cell pellets were treated with lysis buffer composed of 50 mM Tris-HCl pH 8.0 1M (Gibco, Waltham, MA, USA), 10mM UltraPure EDTA 0.5 M, 1% UltraPure SDS (both from Life Technologies, Waltham, MA), supplemented with 1 mg/mL Proteinase K (Ambion, Austin, TX, USA) and incubated overnight at 60 °C. On the next day, to each sample was added phenol/chloroform/isoamyl alcohol (Thermofisher, Waltham, MA, USA), tubes were vortexed for 30 s and centrifuged at room temperature for 10 min. Next, supernatants were collected and DNA was precipitated with 2.5 volumes of 100% EtOH, 0.1 volume of 3 M sodium acetate and 1 μL of glycogen (both from Ambion, Austin, TX, USA) at −20 °C for 1 h. Pellets were washed with 1 mL of ice-cold 75% EtOH, briefly vortexed and centrifuged at 4 °C for 5 min. The supernatant was discarded and pellets were air dried at 60 °C for 5 min. Samples were finally resuspended in H_2_O DNase/RNase free.

### 2.4. PCR Amplification

After DNA extraction, PCR amplification was carried out following the Expand Long Template PCR System protocol (Roche Diagnostic, Mannheim, Germany) as reported in Joensuu et al., 2007 [11]. However, we added some modifications to obtain the best result from our experimental settings. The PCR master mix was prepared in a final volume of 20 μL containing 50 ng of genomic DNA, 1× Expand Long Template Buffer 3, 0.5 mM dNTPs mix, 0.25 μM of each primer, 3.75 U of Expand Long Template Enzyme Mix, 1.0 M GC-Melt Reagent (Takara Bio Inc., Kusatsu, Japan), 10% dimethyl sulfoxide (DMSO), 1.0 M L-Proline (Sigma-Aldrich, St. Louis, MO, USA) and 1× BSA. After an initial denaturation at 94 °C for 2.20 min, 40 cycles were performed at 94 °C for 10 s, 48 °C for 45 s, 68 °C for 7 min and a final elongation at 68 °C for 10 min. For the PCR amplification of the dodecamer sequences were used the following primers: FW (5′-CGCCCGGAAAGACGATACC-3′) and RV (5′-GGCACTTTGGCTTCGGAGT-3′). For the amplification of the *CSTB* gene promoter fragment (−668/−1) we used the following primer pairs: FW (5′-CCACCAGAGAACCCTGCCTTC-3′) and RV (5′-CTTGGCGGCGACGGAGGGAAT-3′). Following the amplification step, PCR products were analysed on 1% agarose gel with 1× loading dye (New England Biolabs, Ipswich, MA, USA) and 100 bp/1 Kb DNA ladders (Solis BioDyne, Tartu, Estonia) to evaluate the length of each fragment.

### 2.5. Sanger Sequencing

For the investigation of coding sequence mutations, the three exons’ regions of *CSTB* gene were PCR amplified using the following primers: exon 1 (FW: 5′-CACGTGACCCCAGCGCCT-3′ and RV: 5′-TAAGGCAGGACTCCGGGCC-3′) and exon 2 (FW: 5′-AAGAAGCCACTGAGACAT-3′ and RV: 5′-TTTCCTACCAGCACCCGTT-3′). Due to its large size, exon 3 was divided into different fragments, that were amplified separately using the following primers: exon 3-A (FW: 5′-GACCTGGAGGGGCGCAGCAA-3′ and RV: 5′-AACACAATGAAATTTAGGA-3′); exon 3-B (FW: 5′-GGATTCTGCAGCTGCTTT-3′ and RV: 5′-TAAAGAGTGGTGGTTAGGA-3′); exon 3-C (FW: 5′-CAGGATTCACACCTGCC-3′ and RV: 5′-TACCTCCCTTTAGAAGCCCA-3′); exon 3-D (FW: 5′-AGGCTTCCCATGGAGCCA-3′ and RV: 5′-ATCACTTTCAAAGCTCTGT-3′); exon 3-E (FW: 5′-GGATCTACCAGTGAGTCCA-3′ and RV: 5′-TACGATCTCGGCTCACTGC-3′); exon 3-F (FW: 5′-GGATCACTTGGACTCGGGA-3′ and RV: 5′-TTCCTGTTGGGGATGGCT-3′); exon 3-G (FW: 5′-TGTTTAGGGGACCACGCA-3′ and RV: 5′-TGTAATTTTGATCCCTTTGT-3′). Exons amplification conditions were carried out according to AmpliTaq Gold™ DNA Polymerase instructions (Thermofisher Scientific, Waltham, MA, USA). PCR program was set as: an initial denaturation at 95 °C for 10 min, 35 cycles at 95 °C for 15 s, 52 °C for 30 s, 72 °C for 30 s and a final elongation at 72 °C for 5 min. For exon 1 the optimal temperature of annealing was at 62 °C for 30 s, for exon 3-A at 45.5 °C, and for exons 3-C and 3-E at 55 °C for 30 s. Gel electrophoresis was performed to confirm the size of each fragment. Next, PCR samples were isolated from agarose gel and purified using QIAQuick Gel Extraction Kit (QIAGEN, Hilden, Germany) and subsequently analyzed by Sanger sequencing (Eurofins Genomics, Ebersberg, Germany).

### 2.6. Fragment Length Analysis by Capillary Electrophoresis

The number of dodecameric repeats in both CTRL and ULD patient’s hiPSCs was determined by fragment length analysis (FLA) by capillary electrophoresis. The PCR amplification of the dodecamer repeat expansion was performed as previously indicated, with 5′-FAM dye-labelled forward primer included in the master mix. PCR products were processed through FLA by capillary electrophoresis analysis by Eurofins Genomics, using GeneScan™ LIZ 1200^®^ Size Standard (Applied Biosystem, Waltham, MA, USA). The data were analysed by Eurofins with GeneMapper™ 6 software (Thermofisher, Waltham, MA, USA).

### 2.7. CSTB Promoter Constructs and Cloning

For the generation of *CSTB* promoter-reporter constructs, a PCR-amplified DNA fragment located −668 bp upstream the transcription start site (from −668 to −1) of *CSTB* gene was cloned into the KpnI and SacI sites (for CTRL) and into the SacI and HindIII sites (for ULD patients) of the promoter-less pGL3-Basic luciferase reporter vector (Promega, Madison, WI, USA), in front of the luciferase reporter gene. Genomic DNA fragments were initially amplified by PCR as described before, using primers incorporating the restriction endonuclease recognition sites. For the cloning of patients’ fragments the following primers were used: FW 5′-GAGCTC-CCACCAGAGAACCCTGCCTTC-3′ including SacI restriction site (underlined), and RV 5′-AAGCTT-CTTGGCGGCGACGGAGGGAAT-3′ including HindIII restriction site (underlined). The primers used to clone healthy control fragment were: FW 5′-GGTACC-CCACCAGAGAACCCTGCCTTCTTC-3′ with KpnI restriction site (underlined) and RV 5′-GAGCTC-TCTCTTGGCGGCGACGGAGGGAATCT with SacI recognition site (underlined). PCR products were loaded on 1% agarose gel without loading dye using TrackIt 1 Kb Plus DNA Ladder (Invitrogen, Waltham, MA, USA). Gel was visualized with Alliance™ Q9-Atom (Uvitec, Cambridge, UK) and single bands were cut, extracted, and purified with QIAquick Gel Extraction kit (QIAGEN, Hilden, Germany) following manufacturer’s procedure. Purified PCR products (CTRL, ULD1-A, ULD1-B, ULD2-A and ULD2-B) and pGL3-Basic vector (Promega, Madison, WI, USA) were double-digested with the relative restriction enzyme pairs (all from New England Biolabs, Ipswich, MA, USA) for 1 h at 37 °C. All samples were then purified with DNA Clean and Concentrator kit (Zymo Research, Irvine, CA, USA) to remove the excess of restriction enzymes and buffer and finally eluted into a proper volume of pre-heated DNase/RNase-free water. The cleaved pGL3-Basic vector was further treated with alkaline phosphatase from the calf intestine (Quick CIP [5U/μL], New England Biolabs, Ipswich, MA, USA). The sample was incubated at 37 °C for 10 min and the reaction was stopped by heat-inactivation at 80 °C for 2 min. To obtain a perfectly pure DNA sample, dephosphorylated pGL3-Basic vector was run on 1% agarose gel without loading dye and band was excised from gel and purified. DNA fragments were incorporated into the vector using T4 DNA Ligase (Invitrogen, Waltham, MA, USA) using a molar ratio of 5:1 (insert to vector). Samples were gently mixed, briefly centrifuged and incubated at room temperature for 5 min. Ligation products were then transformed into Subcloning Efficiency™ DH5α™ Competent Cells (Invitrogen, Waltham, MA, USA) according to the manufacturer’s instructions. After overnight incubation, single colonies were selected and brought up in LB broth medium (Sigma-Aldrich, St. Louis, MO, USA) containing ampicillin (Corning, Corning, NY, USA). Plasmid DNA was extracted using PureLink™ HiPure Plasmid Filter Maxiprep Kit (Invitrogen, Waltham, MA, USA) according to the manufacturer’s protocol. Finally, all promoter-reporter constructs were verified by restriction enzyme digestion to confirm the correct incorporation of inserts into the vector (Figure S1B).

### 2.8. Transfection and Luciferase Assay

For luciferase assay, human 293T cells were seeded in a 24-well plate at the density of 1.5 × 10^5^ cells per well, maintained with Dulbecco’s Modified Essential Medium (DMEM) 1× High Glucose 4.5 g/L implemented with 10% Fetal Bovine Serum (FBS), 1% GlutaMAX™, 1% Sodium Pyruvate and 1% Penicillin/Streptomycin (all from ThermoFisher Scientific, Waltham, MA, USA), and kept into a humidified incubator at 37 °C at 5% CO_2_. When the 70% of confluence was reached, transient transfection was performed using FuGENE^®^ HD Transfection Reagent (Promega, Madison, WI, USA) following the manufacturer’s instructions. Briefly, for each sample the transfection reaction mix was prepared in a final volume of 50 μL as follows: CTRL and patient’s promoter-reporter constructs, pGL3-basic luciferase vector, and FuGENE^®^HD were added to 1× Opti-MEM^®^ medium (Thermofisher Scientific, Waltham, MA, USA) in a ratio of 3:1 (reagent/DNA). pTK-Renilla luciferase plasmid was added in each transfection mix to normalize transfection efficiency. Plus, non-transfected 293T were used as non-treated control (NTC). Each mixture was incubated at room temperature for 15 min, unified to 450 μL of growth medium and finally dispensed on cells which were incubated at 37 °C for 24 h.

Transfected cells were assayed 24h after transfection with the Dual-Luciferase Reporter Assay System kit (Promega, Madison, WI, USA), following the manufacturer’s instructions. Briefly, transfection medium was removed and, after gentle wash in 1× PBS^−/−^, cells were lysed with 1× Passive Lysis Buffer (PLB). After lysis, 100 μL/well of Luciferase Assay Reagent II (LARII) were dispensed into a 96-well white plate to which 20 μL/well of lysate samples were added for assessing luciferase activity. Next, the luciferase reaction was stopped with 100 μL of 1× Stop & Glo Reagent and Renilla luminescence was checked. The assay was carried out on GloMAX^®^-Multi Detection System (Promega, Madison, WI, USA) luminometer with 10s read time for each measurement. The assay was performed on three biological replicates, each with at least three technical repeats. For the analysis, the ratio of firefly and renilla luciferase activity was calculated and each value was compared to that of the empty vector (293T transfected only with the pGL3 basic luciferase vector).

### 2.9. RNA Extraction and Quantitative Real-Time PCR

Total RNA was isolated with TRIzol Reagent (Life Technologies, Carlsbad, CA, USA) and reverse transcribed using the High-Capacity cDNA Reverse Transcription Kit (Applied Biosystems, Waltham, MA, USA). 22 ng of cDNA was processed with SensiFAST SYBR Hi-ROX Kit (Meridian Bioscience, Cincinnati, OH, USA, #BIO-92020) and amplified through qRT-PCR following QuantStudio7 Pro Real-Time PCR System software’s procedure (Applied Biosystems, Waltham, MA, USA) for gene expression quantification. Ct values of each gene were normalized to those of glyceraldehyde 3′-phosphate dehydrogenase (GAPDH), used as a housekeeping gene. A list of the primers used in this study is provided in Table S1.

### 2.10. Western Blotting

For total protein lysis, cells were harvested in 1× PBS^−/−^ and extracted with RIPA Buffer made of 50 mM Tris-HCl 1 M pH 7.5 (Gibco, Waltham, MA, USA), 150 mM Sodium chloride 5 M, 1% Triton X-100, 0.5% Sodium Deoxycholate, 0.1% SDS (all from Sigma-Aldrich, St. Louis, MO, USA) implemented with Halt™ Protease Inhibitors and Halt™ Phosphatase Inhibitors (both from Thermofisher Scientific, Waltham, MA, USA). The protein concentration was determined by Bradford Assay (Bio-Rad, Hercules, CA, USA). Lysates were denatured at 95 °C for 5 min in 1× Laemmli Sample Buffer (Bio-Rad, Hercules, CA, USA). 20 μg of lysates were resolved in Mini-PROTEAN^®^ TGX™ 4–20% Precast Protein Gels and subsequently transferred to a nitrocellulose membrane (both from Bio-Rad, Hercules, CA, USA) using Trans-Blot Turbo transfer system (Bio-Rad, Hercules, CA, USA). Membranes were blocked for 1h at room temperature with 5% milk (PanReac AppliChem, Darmstadt, Germany) in 1× TBS-T and incubated at 4 °C overnight with the following primary antibodies: anti-Cstb (1:250, mouse monoclonal, #MAB1408, R & D System), anti-Cathepsin B (1:500, mouse monoclonal, #ab58802, Abcam), anti-Cathepsin D (1:1500, mouse monoclonal, #ab6313, Abcam), anti-Cathepsin L (1:50, mouse monoclonal, #sc-32320, Santa Cruz) and anti-Parp1 (1:1000, rabbit monoclonal, #9532, Cell Signaling Technology). Anti-Gapdh (1:1000, rabbit polyclonal, #bs-10900R, Bioss Antibodies) was used as a normalizer. After several washes with 1× TBS-T, membranes were incubated with horseradish peroxidase (HRP)-conjugated secondary antibody anti-mouse IgG and anti-rabbit IgG (1:10,000, Jackson ImmunoReasearch, West Grove, PA, USA) for 1h at room temperature. Protein bands were detected through Clarity™ Western ECL Blotting Substrates (Bio-Rad, Hercules, CA, USA) and images were acquired with Alliance™ Q9-Atom (Uvitec, Cambridge, UK). Western blot bands were quantified using the Analyze Gels tool of ImageJ software. A list of the antibodies used in this study is provided in Table S2.

### 2.11. Immunofluorescence Assay

CTRL and ULD patient’s neurons were cultured on Poly-L-ornithine coated (Sigma-Aldrich, St. Louis, MO, USA) round cover glasses and, after 21 days, they were fixed with 4% formaldehyde (Sigma-Aldrich, St. Louis, MO, USA) and blocked with 1% bovine serum albumin (BSA) (Sigma-Aldrich, St. Louis, MO, USA) and 0.1% Triton X-100 (Sigma-Aldrich, St. Louis, MO, USA) in 1× PBS without calcium and magnesium (Corning, Corning, NY, USA). Neurons were immunoassayed with the following primary antibodies: anti-Cstb (1:350, rabbit polyclonal, #HPA017380, Atlas Antibodies, Bromma, Sweden), anti-Cathepsin B (1:200, mouse monoclonal, #ab58802, Abcam, Cambridge, UK), anti-Cathepsin D (1:200, mouse monoclonal, #ab6313, Abcam, Cambridge, UK), anti-Cathepsin L (1:100, mouse monoclonal, #sc-32320, Santa Cruz, Dallas, TX, USA), anti-NEF-H (1:1000, rabbit polyclonal, #ab8135, Abcam, Cambridge, UK), and anti-MAP2 (1:1000, mouse monoclonal, #MA5-12826, Thermofisher Scientific, Waltham, MA, USA) and incubated at 4 °C overnight. The next day, cells were washed with 1× PBS^−/−^ and incubated with AlexaFluor-594 and AlexaFluor-488 conjugated secondary antibodies (1:500, both from Thermofisher Scientific, Waltham, MA, USA) for 1h at room temperature. Nuclei were stained with DAPI (1:800, Carl Roth, Karlsruhe, Germany) and cover glasses were mounted with Dako Fluorescent Mounting Medium (Agilent, Santa Clara, CA, USA). Representative images were acquired with Leica DMi8 inverted microscope and LAS X software (v. 3.7.4.23463; Leica Microsystems CMS GmbH).

### 2.12. Statistical Analysis

All experiments were performed at least on three different biological replicates. Results were analyzed with GraphPad PRISM software, version 9.3.1 (GraphPad Software Inc., San Diego, CA, USA). Data are presented as the mean ± standard error of the mean (SEM). Statistical analysis was performed using two-tailed *t*-test or multiple unpaired *t*-test with Welch correction, with a significance of * *p* ≤ 0.05, ** *p* ≤ 0.01 and *** *p* ≤ 0.001.

## 3. Results

### 3.1. Genetic Characterization of ULD Patients

**Figure 2:**
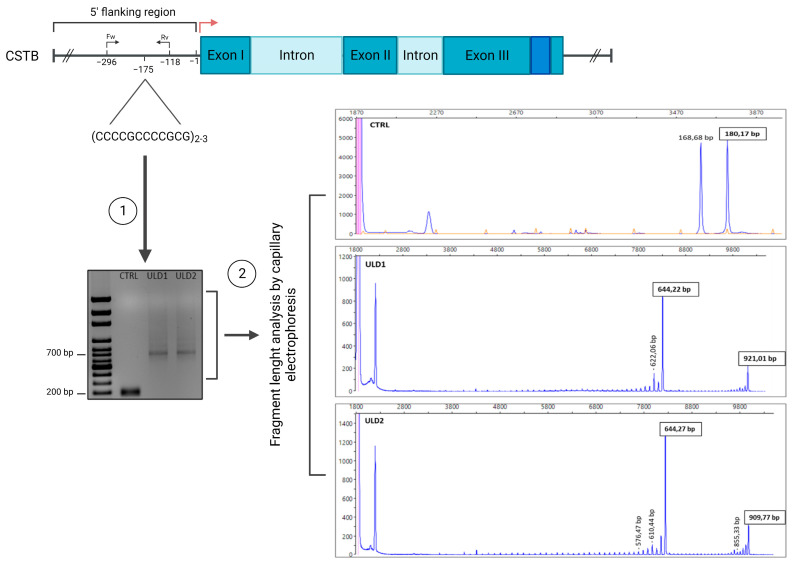
Quantification of the number of dodecamers by fragment length analysis. (**Left**) PCR amplification with 5′-FAM labelled primers of a portion of ~180 bp across the dodecamer repeats of the CSTB promoter, performed on genomic DNA extracted from CTRL and ULD iPSCs and visualization of the amplified fragments on agarose gel (100-bp DNA ladder was used as a size marker). The position of Fw and Rv primers (at −296 bp and −118 bp, respectively) is indicated. (**Right**) Fragment length analysis by capillary electrophoresis of PCR products. The sizes of the main fragments are indicated above the peaks in bp. At 180 bp is the peak for the CTRL (2 dodecamers); at 644 bp are the peaks for the first allele of ULD1 and ULD2 (41 dodecamers); at 921 bp is the peak for the second allele of ULD1 (64 dodecamers); at 909 bp is the peak for the second allele of ULD2 (63 dodecamers). GeneScan LIZ 1200 was used as marker size (orange peaks in CTRL electropherogram) and the data were analyzed by Eurofins with GeneMapper 6 software (Applied Biosystems). Representative images were created using Peak Scanner software (v. 2.0; Applied Biosystems).

ULD is a genetic disease mainly caused by an abnormal expansion of a dodecamer sequence located on the *CSTB* gene promoter. Some patients, especially those with a more severe phenotype, have a compound heterozygous variant of the disease, meaning that one allele carries the dodecamer expansion mutation on the promoter of the *CSTB* gene, while the other allele carries *CSTB* coding sequence point mutations [8,9,10,34]. Sanger sequencing of the entire coding sequence of *CSTB* gene from ULD1 and ULD2 patient’s-derived iPSCs showed no known/unknown point mutations (data not shown), suggesting that both patients were most likely homozygous for the expansion mutation. To detect the exact number of dodecamer repeats in both alleles of the *CSTB* gene in ULD1 and ULD2, we developed a novel PCR-based method coupled with fragment length analysis (FLA) by capillary electrophoresis (see Materials and methods). This PCR method is specific for targeting the amplification of long GC-rich DNA fragments and represents a modified version of the protocol previously developed by Joensuu and colleagues [11]. Furthermore, we included forward primers labelled with 6-FAM fluorescent dye in the PCR amplification mix in order to perform subsequent FLA by capillary electrophoresis, which was crucial to detect the homozygosity and to estimate the precise number of dodecamer repeats in our patients. Genomic DNA was extracted from CTRL and ULD patient’s iPSCs and the PCR protocol was carried out using 5′-FAM labelled primers that amplify a portion of 180 bp (from −293 to −115) across the dodecamer repeats located at 175 bp upstream the transcription start site (TSS) of the *CSTB* gene. This portion carries 2 dodecamers in the wild-type sequence, as confirmed by Sanger sequencing analysis performed on the amplified promoter of the CTRL sample (data not shown). Fragment length analysis of PCR products unequivocally confirmed that both patients are homozygous for the expansion mutation, as shown in the electropherograms in Figure 2, meaning that the dodecamer expansion mutation is present on both alleles. When more than two dodecamers are present, as in the case of our patients, the 180 bp sequence becomes larger in proportion to the number of repetitions present. Indeed, FLA analysis showed that the amplified sequence in the first allele of both patients was 644 bp long, corresponding to the 180 bp sequence with 41 dodecamers instead of two, while for the second allele we found that the ULD1 sequence carries 64 dodecamers (921 bp), while in ULD2 the sequence has 63 repetitions (909 bp). In the control sample, as confirmation of Sanger sequencing, the FLA analysis showed a fragment of 180 bp in both alleles, which means that the healthy control has two dodecamers (Figure 2 and Table 2). This experiment was performed in a second control line that resulted to have 3 dodecameric repeats in Sanger sequencing, obtaining the same result (data not shown).

The fact that both patients with ULD analyzed in this study basically carry the same expansion mutation confirms what is stated in the literature, that is, that there is no correlation between the size of expansion and the age of onset or the clinical severity of the disease [10,12,13]. Therefore, the equivalent entity of the genetic mutation in these two siblings with variable phenotypes of ULD suggests the involvement of additional, yet unknown, molecular events in the onset of the disease.

### 3.2. In Vitro Study of the Effect of Pathological Repeat Expansion on CSTB Promoter Activity

**Figure 3:**
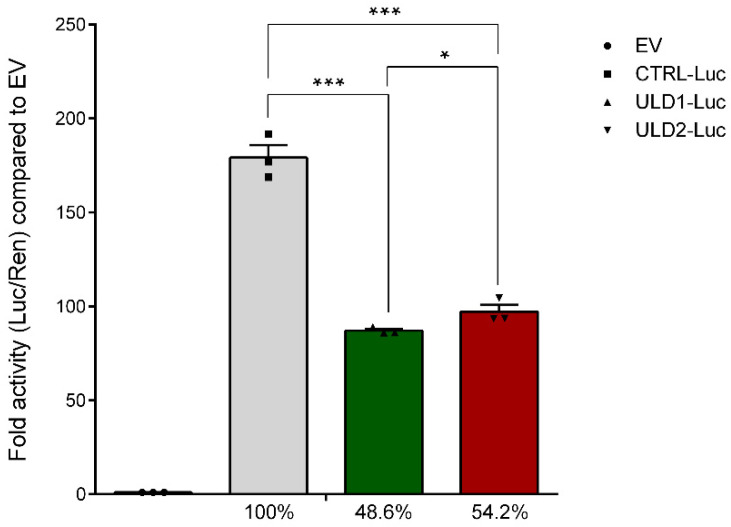
Luciferase reporter assay for CSTB promoter activity assessment after reporter constructs transfection in human 293T cells. ULD patient’s data are plotted in the graph as the mean of activity of the two alleles that were cloned, transfected, and analyzed separately (for the complete result of luciferase assay, see Figure S2). Results from the patient’s constructs show a significantly decreased promoter activity (48.6% for ULD1-Luc and 54.2% for ULD2-Luc) compared to CTRL-Luc, for which the luciferase activity was set at 100%. The ratio of Firefly (Luc) and Renilla (Ren) luciferase activity for each of the three biological repeats (black dots), each with at least three technical replicates, was compared to empty vector (no-promoter pGL3-basic luciferase reporter vector). Data are presented as mean ± SEM, * *p* ≤ 0.05, *** *p* ≤ 0.001, *t*-test has been calculated vs. CTRL-Luc and between ULD1-Luc and ULD2-Luc.

All ULD patients with an expansion mutation in the *CSTB* promoter region show a decreased transcription rate of the *CSTB* gene and a reduced *CSTB* promoter activity [11]. Among all the human neurodegenerative diseases caused by expansion mutations, ULD is the only one that presents a repeat unit in the promoter region. Previous in vitro studies on *CSTB* promoter have shown that the unstable expansion of dodecamer repeats (>30) significantly reduces promoter activity [35,36] and, consequently, leads to a drastic downregulation of *CSTB* gene and protein expression [12]. In the present work, we have analyzed the effect of the expansion mutation on the *CSTB* promoter activity of two patients with ULD in vitro, using reporter gene constructs. In 2000, Alakurtti et al. reported that the dodecamer repeat is located about 175 bp upstream of the TSS in the *CSTB* promoter region [35]. Based on this, we designed pairs of PCR primers with endonuclease restriction sites for the amplification and subsequent cloning of a ~670 bp fragment upstream the TSS (from −668 to −1) of the promoter of the *CSTB* gene. Although previous studies [35,36] failed to directly clone the longer repeat expansion fragments derived from homozygous patients into luciferase-based vectors, in this work we succeeded to amplify and insert the *CSTB* promoter fragments derived from the two ULD patients into the promoter-less pGL3-basic luciferase reporter vector. The PCR amplification of the *CSTB* promoter resulted in a single fragment of 668 bp for CTRL, while for both patients, we obtained two fragments: 1172 bp and 1447 bp for ULD1, and 1171 bp and 1435 bp for ULD2 (Figure S1A). These two fragments correspond to the two alleles of the *CSTB* gene that, consistent with our FLA results, have a different size due to the presence of a diverse number of dodecamer repeats. Therefore, to obtain accurate promoter activity, we sought to clone and analyze them separately. After cloning, the obtained recombinant plasmids (namely: CTRL-Luc, ULD1-A-Luc, ULD1-B-Luc, ULD2-A-Luc, and ULD2-B-Luc) were transiently transfected in human 293T cells and luciferase assay was performed to estimate *CSTB* promoter activity. Following this strategy, we obtain the result that the four promoter constructs of the patients showed significantly lower activity in terms of luciferase expression, compared to CTRL-Luc (Figure S2). Specifically, the constructs derived from the smaller alleles, ULD1-A-Luc and ULD2-A-Luc, showed a 36.4% and 27.7% of activity, respectively, compared to control, while the other two construct containing the fragment derived from the alleles with more dodecamers displayed a luciferase expression of 60.8% for ULD1-B-Luc and 80.8% for ULD2-B-Luc. Furthermore, we calculated the mean activity of the two constructs of each patient. As a result, the luciferase expression of normal promoter construct (CTRL-Luc), containing two dodecamer repeats, resulted to be ~180-fold higher compared to the empty vector (pGL3-basic), while the constructs containing pathological repeat expansions (ULD1-Luc and ULD2-Luc) showed a two-fold reduction of luciferase expression compared to normal promoter construct (48.6–54.2%, Figure 3), therefore confirming an overall significant reduction of *CSTB* gene promoter activity in ULD1 and ULD2 patients compared to that of the healthy subject.

### 3.3. Assessment of Cystatin B Expression in ULD Patient-Derived iPSCs and Neurons

**Figure 4:**
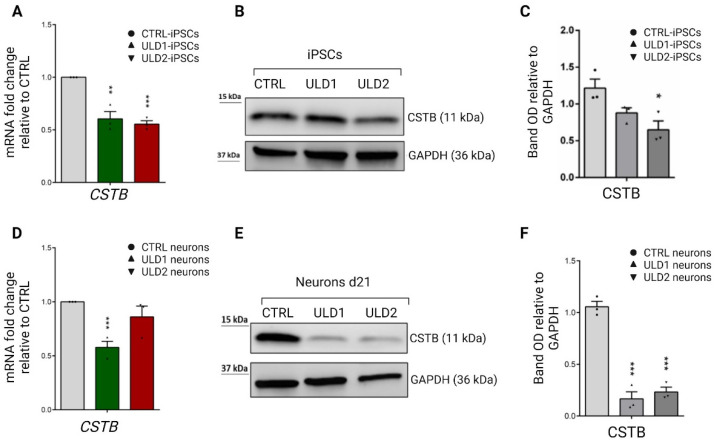
Expression of CSTB gene and protein in iPSCs and neurons derived from CTRL and ULD patients. (Top panel) (**A**) qRT-PCR analysis of CSTB gene in iPSCs. ULD patient-specific iPSCs show a significantly lower expression of CSTB compared to CTRL iPSCs. (**B**) Western blot analysis of CSTB protein in total lysates obtained from CTRL, ULD1, and ULD2 iPSCs. (**C**) Densitometric analysis of WB results, performed using ImageJ software. (Bottom panel) (**D**) qRT-PCR analysis of CSTB gene in neurons at day 21 of differentiation. ULD patient-derived neurons, particularly ULD1, show a lower expression of CSTB compared to CTRL neurons. (**E**) Western blot analysis of CSTB protein in total lysates obtained from CTRL, ULD1, and ULD2 neurons show a significant decrease of CSTB protein expression in patient’s cells compared to control. (**F**) Densitometric analysis of WB results, performed using ImageJ software. In all WB analyses GAPDH was used as loading control. Both qRT-PCR and WB data are presented as mean ± SEM of three independent experiments (black dots), * *p* ≤ 0.05, ** *p* ≤ 0.01, *** *p* ≤ 0.001. For neurons, *t*-test has been calculated vs. CTRL at the same day of differentiation.

Many clinical and molecular studies conducted in patients with ULD homozygous for the expansion mutation reported a marked reduction in steady-state expression levels of the *CSTB* gene and protein [7,8,11,37]. These data are in line with the in vitro studies on *CSTB* promoter activity, including the present work, which have shown a significant decrease in promoter activity in the presence of an abnormal expansion of the dodecamer repeat sequence [35,36]. It still remains unclear how this type of promoter mutation leads to the drastic drop—but not to complete loss—of cystatin B synthesis, which is followed by the reduction of CSTB protein expression [11,16] and, consequently, of its inhibitory activity [14]. A possible hypothesis to explain the down-regulation of *CSTB* transcription in homozygous patients is that the dodecamer expansion could alter the spacing of promoter elements from the transcription initiation site, perhaps through the formation of tetraplex secondary structures, thus disturbing transcriptional machinery [12,35]. Based on these premises, we sought to evaluate the expression levels of cystatin B mRNA and protein in CTRL and ULD in the patient’s iPSCs and iPSCs-derived neurons. Compatible with our in vitro promoter studies, showing a reduction in *CSTB* promoter activity in patients, the *CSTB* mRNA expression level was down-regulated in the patient’s iPSCs and neurons, compared to the healthy control (Figure 4A,D). Accordingly, western blot analysis revealed a decreased expression of CSTB protein in the ULD patient’s iPSCs compared to control (Figure 4B,C). Intriguingly, we observed a much more evident reduction in the expression of the CSTB protein in neurons differentiated from ULD iPSCs, where the expression of CSTB protein in ULD1 and ULD2 neurons resulted in −90.3% and −83.5%, respectively, compared to control neurons (Figure 4E,F)

The reduced expression of the CSTB protein in neurons from the ULD patients with respect to CTRL neurons was further confirmed by immunofluorescence analysis shown in Figure S4A. Although there is clear evidence that the size of the expansion repeat does not correlate with the severity of the disease or the age of onset, a low level of CSTB expression was shown to be associated with a more severe pathological phenotype [38]. Accordingly, we found a lower expression of the CSTB protein in patient ULD1, which shows a more severe phenotype of the disease than in patient ULD2, suggesting that the clinical severity is inversely proportional to the residual amount of the CSTB protein. This aspect is of particular interest to us since the two siblings analyzed in this study bear the same genetic mutation, but show different disease phenotypes. However, further investigations are needed to fully understand how reduced expression levels of cystatin B can be responsible for different degrees of severity in the presence of the same genetic mutation, as in the case of the two siblings in our study.

### 3.4. Assessment of Cathepsins Expression in ULD Patient-Derived iPSCs and Neurons

**Figure 5:**
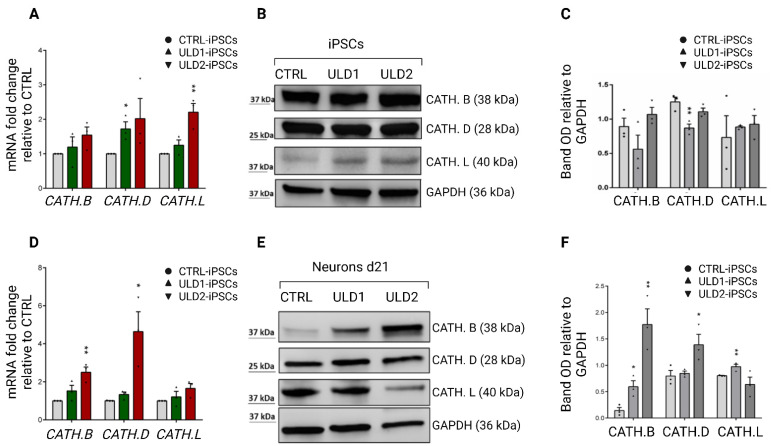
Gene and protein expression analysis of cathepsins B, D and L in iPSCs and neurons derived from CTRL and ULD patients. (Top panel) (**A**) qRT-PCR analysis of cathepsins mRNA expression in iPSCs. ULD patient-derived iPSCs show a higher expression of cathepsins compared to CTRL iPSCs. (**B**) Western blot analysis of total lysates obtained from CTRL and ULD iPSCs does not show an enhanced cathepsins expression in patients iPSCs compared to control. (**C**) Densitometric analysis of WB results, performed using ImageJ software. (Bottom panel) (**D**) qRT-PCR analysis of cathepsins mRNA expression in neurons at day 21 of differentiation. ULD patient-derived neurons show a higher expression of cathepsins genes compared to control. (**E**) Western blot analysis of total lysates obtained from CTRL and ULD neurons results in an overall increase of cathepsins protein expression (particularly cathepsins B and D for ULD2) in patients compared to control, (**F**) Densitometric analysis of WB results, performed using ImageJ software. In all WB analyses, GAPDH was used as loading control. qRT-PCR and WB data are each presented as mean ± SEM of three independent experiments (black dots), * *p* ≤ 0.05, ** *p* ≤ 0.01. For neurons, *t*-test has been calculated vs. CTRL at the same day of differentiation.

The main physiological role of cystatin B concerns the inhibition of several lysosomal cysteine proteases belonging to the cathepsin family [39,40,41]. This inhibition occurs through the conserved QVVAG amino acid sequence of CSTB, which represents the catalytic site responsible for interaction with cathepsins, particularly B, L, and S [14,42,43]. Cathepsins are proteases involved in intralysosomal protein degradation and in a wide range of biological processes, including antigen processing, oxidative stress, and apoptosis [14,44]. Although the loss of inhibition by CSTB on cathepsins is recognized as the main cause of the ULD, it is not yet clear what are the molecular mechanisms by which cystatin B is able to inhibit these proteases [20]. CSTB deficiency was shown to correlate with enhanced expression of cathepsins, both in vivo and in vitro [14]. On this basis, we sought to evaluate the expression levels of three cathepsins, B, D, and L, in iPSCs and neurons differentiated from ULD and healthy control subjects. Not surprisingly, diseased cells which show a reduction of cystatin B expression, both at mRNA and protein levels, displayed an overall tendency to express higher levels of cathepsins compared to control cells. In particular, we found a higher expression of cathepsin mRNA in iPSCs from patients with ULD (Figure 5A), while at the protein level their expression was not significantly modulated (Figure 5B,C). On the other hand, both protease mRNA and protein expression resulted up-regulated in neurons differentiated from patients compared to those derived from control iPSCs (Figure 5D–F), therefore confirming that a lower expression of cystatin B is associated with an enhanced expression of cathepsins and that this condition only becomes apparent in mature cells.

Furthermore, the expression of cathepsins B, D, and L in CTRL and the ULD patient’s neurons by immunofluorescence analysis confirmed an increased expression in diseased cells (Figure S4B–D). Interestingly, the western blot analysis of neurons revealed that cathepsins B and D are significantly more expressed in ULD2, which is affected by a milder pathological phenotype, while ULD1 displays only a slightly higher, statistically significant, expression of cathepsin L compared to ULD2. This data suggests that the pathological function of mutated cystatin B is likely not limited to cathepsin regulation, and that other molecular mechanisms need to be taken into account in order to discriminate different degrees of ULD severity.

### 3.5. Investigation of Cathepsins-Mediated Apoptosis in ULD-Neurons

**Figure 6:**
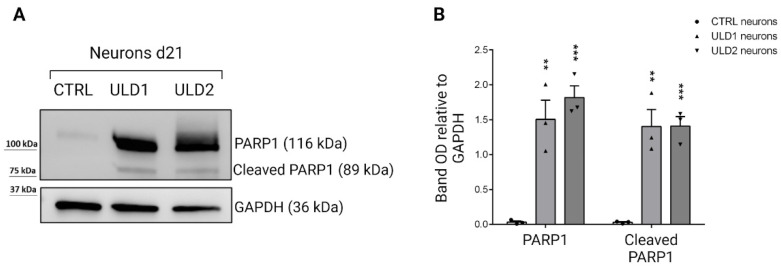
Protein expression analysis of PARP1 in neurons derived from CTRL and ULD patients. (**A**) Western blot analysis of total lysates obtained from CTRL and ULD neurons results in high level of expression of full length PARP1 in patients compared to control; the expression of 89-kDa fragment produced by the cleavage of PARP1 is only detectable in patient’s cells. (**B**) Densitometric analysis of WB results, performed using ImageJ software. GAPDH was used as loading control. Data are presented as mean ± SEM of three independent experiments (black dots), ** *p* ≤ 0.01, *** *p* ≤ 0.001. *t*-test has been calculated vs. CTRL neurons at the same day of differentiation.

Programmed cell death, or apoptosis, can be induced by diverse intracellular events through two main signaling pathways: the extrinsic or death receptors pathway, and the intrinsic or mitochondrial pathway. Both require, from their early stages, the action of a variety of proteolytic enzymes, including caspases, calpains, matrix metalloproteinases, and lysosomal cathepsins [45,46]. There is much evidence that, in response to apoptotic stimuli, cathepsin proteases are released from lysosomes into the cytosol and participate in cell death programs [47] in different ways: by triggering the mitochondrial pathway via the cleavage of the pro-apoptotic Bcl-2 family member Bid [47,48], or by directly cleaving key cellular substrates, such as PARP1 (poly(ADP-ribose) polymerase) [49]. In 2010, Chaitanya and colleagues reported that, in addition to caspases, cathepsins also exert, in response to apoptotic stimuli, their proteolytic activity on PARP1 producing several cleavage fragments. Specifically, cathepsins B and D can produce the 89-kDa PARP-1 fragment, which is a known marker of apoptotic cell death [30]. Therefore, our objective was to investigate the presence of this specific cathepsin-mediated apoptotic mechanism in the neurons of our ULD patients. Western blot analysis showed that ULD1 and ULD2 neuron cells show higher levels of the steady-state expression of full-length PARP1 compared to the control, and interestingly, the 89-kD fragment of cleaved PARP1 is produced only in patients (Figure 6). This result suggests that, in individuals affected by ULD, cathepsins may mediate neuronal apoptosis through PARP1 cleavage.

## 4. Discussion and Conclusions

The cystatin B (or Stefin B) gene is located on chromosome 21q22.3 and encodes a 98 amino acid protein, which is a ubiquitously expressed member of a superfamily of protease inhibitors [7,40,50,51]. In 1996, Pennacchio and colleagues identified the *CSTB* gene as responsible for Unverricht-Lundborg disease (ULD, or EPM-1), an autosomal recessive inherited form of progressive myoclonus epilepsy [7]. So far, at least 15 mutations in the *CSTB* gene have been identified as causal to disease [11,12,34,52,53]. A total of 90% of ULD patients are homozygous for the abnormal expansion mutation of the dodecamer sequence located in the *CSTB* gene promoter, while a small percentage of patients are compound heterozygous, thus bearing point mutations in the *CSTB* gene coding sequence in addition to the repeat expansion [8,9,10,34]. This group of patients normally shows a more severe disease phenotype. Unlike other disorders caused by repeat mutations in non-coding region, e.g., Fragile X syndrome, Friedreich ataxia, Myotonic dystrophy, and Spinocerebellar ataxias, in which the repeated sequence is always a trinucleotide, ULD is the only disease so far known to be caused by the expansion of a dodecamer sequence in the gene promoter region [16,54]. The homozygous dodecamer expansion mutation leads to a reduced expression of the *CSTB* gene and protein, thus impairing its inhibitory activity [14]. It is widely accepted that the size of expansion mutation does not correlate with the disease severity nor with its age of onset [10,13]. In this study, to characterize the genetic mutation of the disease, we reprogrammed fibroblasts and T lymphocytes from two Italian siblings suffering from Unverricht-Lundborg disease, ULD1 and ULD2, to generate induced pluripotent stem cells (iPSCs). Patient-specific iPSCs provide an unlimited source of disease-relevant cells and represent a robust cellular platform for a plethora of biological applications, including in vitro model disease, drug screening, and testing, as well as holding the potential for clinical applications [55,56,57,58,59]. The vast majority of genetic disorders have different effects in different people and this holds true even when comparing the siblings examined in this study as they show different degrees of disease severity: ULD1 has a severe phenotype, while ULD2 has a mild form. To investigate in this direction and understand the molecular differences underlying the disease phenotype, we characterized the promoter of the *CSTB* gene of both patients and tried to define how specific mutations can discriminate and thus explain the different disease phenotypes. First, we asked whether they were homozygous for dodecamer repeats or compound heterozygous carrying mutations in the *CSTB* coding sequence region. The presence of dodecamer repeats expansion makes it impossible to perform direct sequencing analysis for number of repeats quantification [4], due to the impressive GC content. Southern blotting or PCR amplification for long GC-rich DNA templates coupled to capillary electrophoresis are the methods of choice [11]. Here, we have devised a long GC-rich PCR-based method paired with fragment length analysis (FLA) by capillary electrophoresis to quantify the number of dodecamer repeats present in the patient’s *CSTB* gene promoter. Our results show that ULD1 and ULD2 are homozygous for expansion mutation and, despite having a severe and a mild form of the disease, respectively, they share the same number of dodecamer repeats on both alleles of *CSTB* gene. This, together with the fact that the age of onset of the disease is the same for both patients, is in line with previous data showing that the severity of the disease does not necessarily correlate with the size of alleles, in terms of dodecamer repeats, and that other events (molecular, genetic, or epigenetic mechanisms) may be involved in the diversity of phenotypes. To further investigate this direction, we performed an in vitro characterization of the promoter activity of the *CSTB* gene. Cloning the *CSTB* gene promoter fragments from a homozygous patient containing more than 50 repeats into luciferase plasmids is known to be extremely challenging [35,36]. Here, to our knowledge for the first time, we have succeeded in assembling the amplified fragments derived from the ULD1 and ULD2 *CSTB* promoter into the pGL3-basic luciferase reporter vector. As expected, since the two patients share the same expansion mutation of the dodecamer, the promoter activity resulted to be the same, but reduced by two-fold compared to the healthy control line. The abnormal expansion of the dodecamer sequence disrupts the transcriptional machinery in the promoter, causing a down-regulation of *CSTB* mRNA and protein expression [11,20,34]. An impaired transcription may be due to an alteration of the transcription binding sites from the translation initiation codon either caused by repeat expansion [36], or disruption of DNA methylation status [60], or the formation of tetraplex structures typically formed by repeat sequences containing guanine and cytosine residues [61]. An analysis of cystatin B expression in patients with ULD resulted in a downregulation of mRNA and protein levels, both in iPSCs and neurons, for which the reduction of CSTB protein was even more significant. Interestingly, in patient ULD1 (severe) the expression level of CSTB protein resulted slightly lower than what we could observe for patient ULD2, suggesting that the severity of disease might be somehow dependent on the residual amount of CSTB. As the main physiological role of cystatin B is to inhibit cysteine proteases, a cystatin B deficiency results in an increase in cathepsin activity and, thus, in a dysregulation of intracellular proteolysis [14]. In vitro, the interaction between CSTB and cathepsins has been demonstrated to occur through a catalytic site made up of the highly conserved Gln-Val-Val-Ala-Gly (QVVAG) sequence and the glycine at position 4 of CSTB [15,62]. Among the most studied cathepsins are the cysteine proteases of the papain-like family, comprising cathepsins B, H, L, S, and the aspartic cathepsins of the pepsin-like family, to which cathepsins D and E belong. Each of these enzymes have highly specific proteolytic activity, which is optimal at distinct pH conditions and is essential for the maintenance of homeostasis and for the regulation of many biological processes [63]. Cysteine cathepsins are predominantly located in endosome/lysosome compartments and are involved in many cellular processes, such as protein turnover, apoptosis, autophagy, antigen processing, immune response, and prohormones processing. A dysregulation of these proteases leads to a variety of pathologies: cardiovascular and neurodegenerative diseases, cancer, and arthritis [63,64,65,66]. Aspartic cathepsins are lysosomal proteases that can be strongly inhibited by pepstatin A and, when dysregulated, contribute to the invasive and metastatic potential of the cancer cells [67]. In 2002, Rinne and co-workers found that cells from ULD patients with decreased CSTB activity display an enhanced activity of cathepsins B, L, and S [14] demonstrating, for the first time, the in vivo regulation of cathepsins by cystatin B. Noteworthy, the levels of cathepsin D were found to be increased in the brain of cystatin B-KO mice [68] and it was shown to inactivate cystatin B, thereby enhancing the proteolytic activity of cysteine cathepsins, particularly cathepsin B [69,70]. Based on these considerations, we sought to measure the expression levels of mRNA and protein of cathepsins B, D, and L in neurons derived from the patient’s iPSCs, where the level of cystatin B was shown to be reduced compared to control cells. Our results show an increased expression of cathepsins in the patient’s cells, and this increase in expression was more evident in differentiated neuronal cells. In particular, the severe patient (ULD1) had more cathepsins B and L, while the mild patient (ULD2) displayed higher levels of cathepsins B and D. There is growing evidence that cathepsin proteases can trigger cell-specific apoptosis [71,72,73]. In response to specific signals, cathepsins are released from lysosomes and initiate apoptosis either by activating the caspases-mediated pathway or by acting on the pro-apoptotic Bcl-2 family [74]. In vivo studies on CSTB knockout mice models and ULD patients expressing low levels of cystatin B have shown a loss of cerebellar granule cells [18] and a reduction of the pool of cortical GABA interneurons [20,75] due to apoptotic death; this causes an imbalance of excitatory/inhibitory neurons, favoring seizures and myoclonic events. It is still unclear how the deficiency of cystatin B expression can lead to neuronal cell apoptosis. Interestingly, based on observations of massive apoptotic death of cerebellar granule cells in the CSTB-deficient mouse model [18], Houseweart and colleagues demonstrated that CSTB-Cathepsin B double-deficient mice display a reduced number of dead granule cells [74], strengthening the role that cathepsins play in inducing apoptosis of neurons following a reduction in physiological levels of cystatin B. In the same year, Houseweart et al. showed that cathepsins-mediated apoptosis in ULD was not dependent on proapoptotic Bcl-2 family member Bid [71], suggesting that these proteases may be involved in different cell death signaling. Here, we hypothesized that the loss of cystatin B and the consequent uncontrolled activation of cathepsins could have an effect on apoptosis through the involvement of the cleaved PARP1 (poly [ADP-ribose] polymerase 1), a marker of cells undergoing apoptosis. Indeed, in addition to being a substrate for several caspases, PARP1 was also reported to be a target for cathepsin proteases [30,49]. It was shown that activated cathepsins B and D can cleave PARP-1 producing active fragments of 55-kDa, 42-kDa, and 89-kDa, the latter specifically detectable during apoptotic cell death [30]. Additionally, it was demonstrated that cathepsin L participates in the activation of caspase-3 [76], that is one of the main caspases that cleave PARP-1 to mediate programmed cell death. Based on this, we investigated the presence of the 89 kDa fragment resulting from PARP-1 cathepsin cleavage in the ULD patient’s-derived neurons. We found that patients express significantly higher levels of full-length PARP1 compared to the control and that the 89 kDa fragment of cleaved PARP1 is produced only in ULD1 and ULD2 neurons, which express high levels of cathepsins. Altogether, in addition to having characterized for the first time and in great detail the role of dodecameric repeats in patients with ULD, our data shed some light on the pathogenesis of ULD, showing that in the patient’s cells, cathepsins may be responsible for mediating neuronal apoptosis through the cleavage of PARP1. However, since the levels of the cleaved PARP1 protein are similar in the two patients tested, it is evident that this mechanism cannot explain the phenotypic difference. It is known that CSTB compound heterozygous mutations are responsible for a more severe phenotype, but the phenotypic variability is also present between individuals with the same homozygous CSTB mutation, as in the case of the present study. Phenotype variations in many forms of rare inherited diseases with a known disease-causing variant have been linked to the influence of modifier genes, i.e., independent genetic variants that can modulate disease severity and penetrance. To date, no genetic modifiers have been associated to CSTB and Unverricht-Lundborg disease pathophysiology. A more in-depth study of potential genetic modifiers, as well as of other molecular and epigenetic events, is fundamental to detect the cause of phenotypic variability in ULD patients harboring the same genetic mutation.

## Figures and Tables

**Table 1:** Summary of the clinical features, treatment and genetic mutation of ULD1 and ULD2 patients.

Patient	Gender	Age	Severity	Myoclonus	Epilepsy	Dysarthria	Photosensitivity	Cognitive Impairment	Pharmacological Treatment	CSTB Mutation
ULD1	M	11	Severe	Yes	Yes	Yes	No	Yes	VPA, LEV, PB, CLN	Homozygous for dodecamer repeat
ULD2	F	12	Mild	Yes	Yes	No	No	No	VPA, LEV, PB	Homozygous for dodecamer repeat

VPA: Valproate; LEV: Levetiracetam; PB: Phenobarbitone; CLN: Clonazepam.

**Table 2:** Summary of data obtained with FLA.

Sample name	Fragments Size		N of Dodecamers	
	First allele	Second allele	First allele	Second allele
CTRL-iPSCs	180 bp	180 bp	2	2
ULD1-iPSCs	644.22 bp	921.02 bp	41	64
ULD2-iPSCs	644.27 bp	909.77 bp	41	63

## Data Availability

All the data presented in this study are available from the corresponding author upon reasonable request.

## Supplementary Materials

The following supporting information can be downloaded at: https://www.mdpi.com/article/10.3390/cells11213491/s1, Figure S1: Visualization of CTRL and ULD patient’s CSTB promoter amplified fragments (A) and verification of recombinant vectors after cloning (B); Figure S2: Schematic structure of CSTB promoter-reporter constructs (A) and complete luciferase reporter assay for CSTB promoter activity assessment (B); Figure S3: Characterization of CTRL and ULD patient’s iPSC-derived neurons (q-RT-PCR); Figure S4: Representative immunofluorescence images of CSTB (A) and cathepsins B, D and L (B, C, D, respectively) expression in CTRL and ULD patient’s neurons; Table S1: list of primers; Table S2: list of antibodies.
