# Supplementary material for: Insights into the Genetic Profile of Two Siblings Affected by Unverricht-Lundborg Disease Using Patient-Derived hiPSCs
Source: Cells. 2022 Nov 4;11(21):3491. doi: 10.3390/cells11213491 (PMC9655992; doi:10.3390/cells11213491)
Supplement: Supplementary file 1 [file cells-11-03491-s001.zip › cells-1971365-supplementary.pdf]

SUPPLEMENTARY FIGURES + CAPTIONS:

Figure S1

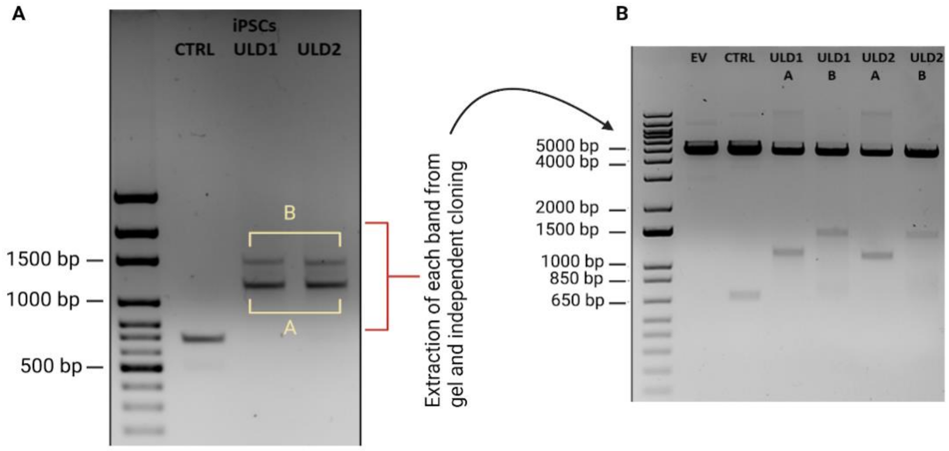

Figure S1. (**A**) Agarose gel showing CTRL and ULD patient's promoter fragments amplified by PCR. The doublet band in ULD1 and ULD2 indicate the two alleles, having a different size corresponding to the different number of dodecamer repeats (See Table 2). The band of CTRL and the two bands of each patient were individually extracted from gel to be cloned separately. 100 bp DNA ladder was used as marker size. (**B**) Verification of CTRL and ULD recombinant vectors by restriction enzyme digestion after cloning. Empty vector (EV) and CTRL promoter vector were double digested with KPNI/SACI and ULD promoter vectors were double digested with SACI/HINDIII and analysed by agarose gel electrophoresis. (Expected inserts size: EV (pGL3-basic): 4818 bp; CTRL: 668 bp; ULD1 – Allele A: 1172 bp; ULD1 – Allele B: 1447 bp; ULD2 – Allele A: 1171 bp; ULD2 – Allele B: 1435 bp). As a size marker, 100 bp (A) and 1Kb DNA ladder (B) were used.

Figure S2

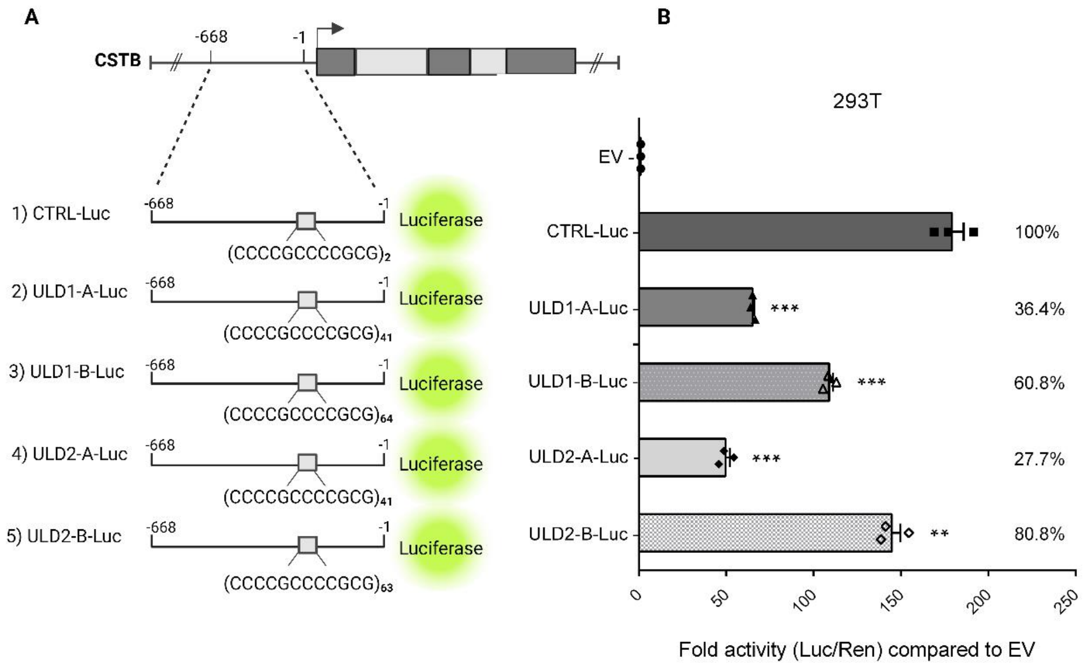

Figure S2. Complete luciferase reporter assay for CSTB promoter activity assessment. (**A**) CSTB promoter constructs (1-5) cloned into promoter less pGL3-basic luciferase reporter vector (EV). Construct 1 (CTRL-Luc) contains wild type CSTB promoter sequence from -668 to -1 with 2 dodecamer repeats. Constructs from 2 to 4 contain the same CSTB promoter region derived from two ULD patients: ULD1-A-Luc and ULD2-A-Luc contain 41 and 64 dodecamers, respectively, of ULD1 patient's CSTB promoter, while ULD2-A-Luc and ULD2-B-Luc contain 41 and 63 dodecamers, respectively, of ULD2 patient's CSTB promoter. This figure was created using Biorender.com. (**B**) Luciferase activity was measured for each of the CSTB promoter-reporter constructs, after they were individually cloned and transfected in human 293T cells. Results for the patient's constructs show an overall significantly decreased promoter activity compared to that of CTRL-Luc, which was set at 100%. The ratio of Firefly (Luc) and Renilla (Ren) luciferase activity for each of three biological repeats (dots), each with at least three technical replicates, was compared to the empty vector. Data are presented as mean ± SEM, ** $p \leq 0.01$, *** $p \leq 0.001$, t-test has been calculated vs. CTRL-Luc.

Figure S3

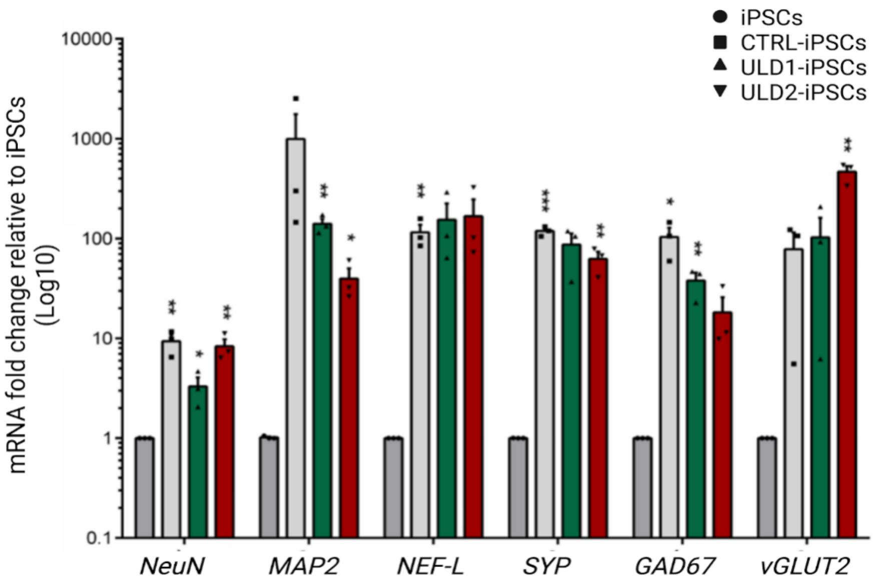

Figure S3. Characterization of CTRL and ULD patient's iPSC-derived neurons. qRT-PCR analysis shows that control- and patients-derived neurons express higher levels of expression of specific neuronal genes such as *NeuN*, *MAP2*, *NEF-L*, *SYP*, *GAD67* and *vGLUT2* compared to their undifferentiated counterparts (iPSCs). Data are presented as mean ± SEM of three biological replicates (black dots), * $p \leq 0.05$, ** $p \leq 0.01$, *** $p \leq 0.001$, t-test has been calculated between each neuronal cell line and the corresponding iPSC line.

Figure S4

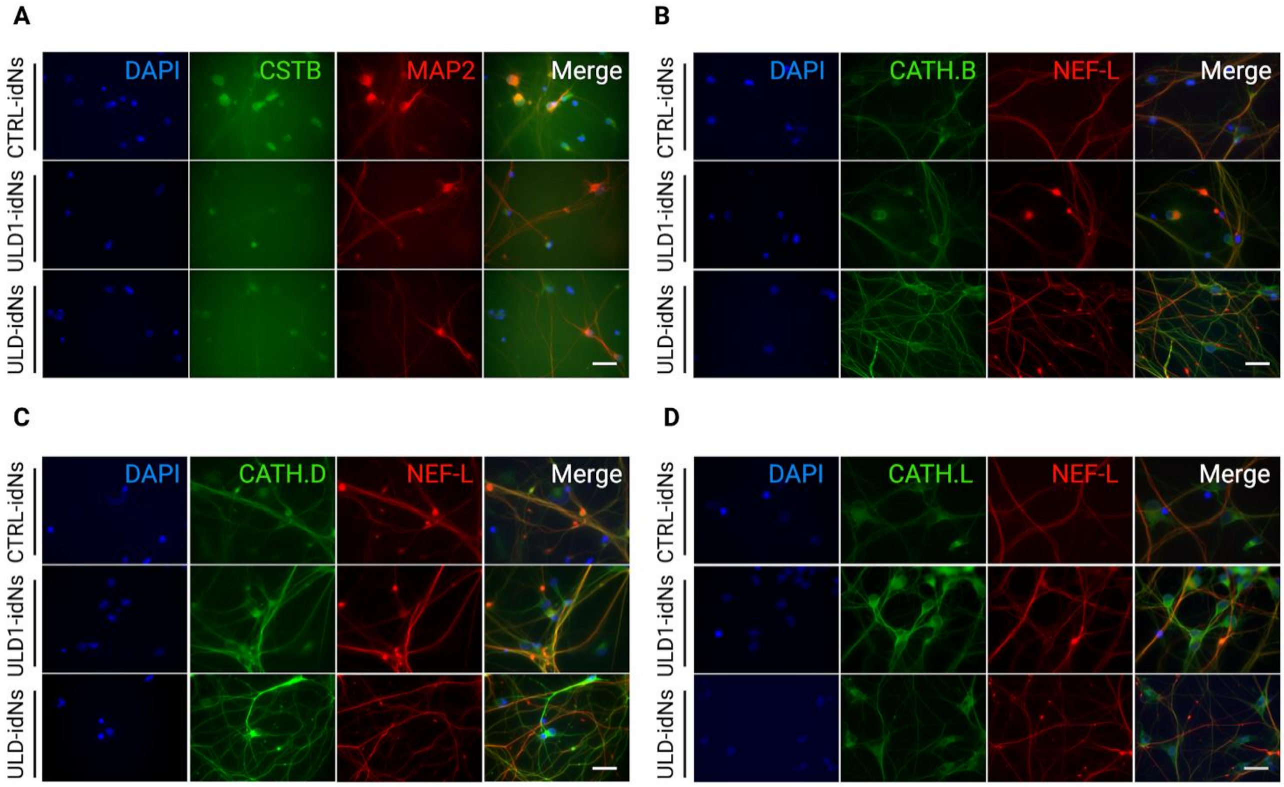

Figure S4. Representative immunofluorescence images of CSTB (**A**) and cathepsins B, D and L (**B**, **C**, **D**, respectively) expression in CTRL and ULD patient's neurons. CSTB and cathepsins were co-stained with MAP2 and NEF-L, respectively, which are markers of mature neurons. CSTB results to be less expressed in ULD-neurons compared to control, and cathepsins expression reflects that of western blot analysis (see Figure 5E,F) (63X magnification, scale bar: 25 µm).

TABLE S1: LIST OF PRIMERS

Table S1. List of primers.

Target Gene		Primer sequence
CSTB Dodecamer	Forward	5’-CGCCCGGAAAGACGATACC-3’
	Reverse	5’-GGCACTTTGGCTTCGGAGT-3’
CSTB Dodecamer 5’-FAM	Forward	5’- CGCCCGGAAAGACGATACC-3’
CSTB Promoter	Forward	5’-CCACCAGAGAACCCTGCCTTC-3’
	Reverse	5’-CTTGGCGGCGACGGAGGGAAT-3’
CSTB Exon 1	Forward	5’- CACGTGACCCCAGCGCCT-3’
	Reverse	5’- TAAGGCAGGACTCCGGGCC-3’
CSTB Exon 2	Forward	5’-AAGAAGCCACTGAGACAT-3’
	Reverse	5’-TTTCCTACCAGCACCCGTT-3’
CSTB Exon 3A	Forward	5’-GACCTGGAGGGGCGCAGCAA-3’
	Reverse	5’-AACACAATGAAATTTAGGA-3’
CSTB Exon 3B	Forward	5’-GGATTCTGCAGCTGCTTT-3’
	Reverse	5’- TAAAGAGTGGTGGTTAGGA-3’
CSTB Exon 3C	Forward	5’-CAGGATTCACACCTGCC-3’
	Reverse	5’-TACCTCCCTTTAGAAGCCCA-3’
CSTB Exon 3D	Forward	5’-AGGCTTCCCATGGAGCCA-3’
	Reverse	5’-ATCACTTTCAAAGCTCTGT-3’
CSTB Exon 3E	Forward	5’-GGATCTACCAGTGAGTCCA-3’
	Reverse	5’- TACGATCTCGGCTCACTGC-3’
CSTB Exon 3F	Forward	5’-GGATCACTTGGACTCGGGA-3’
	Reverse	5’-TTCCTGTTGGGGATGGCT-3’
CSTB Exon 3G	Forward	5’- TGTTTAGGGGACCACGCA-3’
	Reverse	5’- TGTAATTTTGATCCCTTTGT-3’
CSTB Promoter SacI and HindIII	Forward	5’-GAGCTCCCACCAGAGAACCCTGCCTTC-3’
	Reverse	5’-AAGCTTCTTGGCGGCGACGGAGGGAAT-3’
CSTB Promoter KpnI and SacI	Forward	5’-GGTACCCCACCAGAGAACCCTGCCTTCTTC-3’
	Reverse	5’-GAGTCTCTTGGCGGCGACGGAGGGAATCT-3’
CSTB	Forward	5’-TCCCTGTGTTTAAGGCCGTG-3’
	Reverse	5’- GCTTGGCTTTGTTGGTCTGG-3’
CATHEPSIN B	Forward	5’-CTGTGTATTCGGACTTCCTGC-3’
	Reverse	5’-CCAGGAGTTGGCAACCAG-3’
CATHEPSIN D	Forward	5’-AACTGCTGGACATCGCTTG-3’
	Reverse	5’-AGGTACCCGGAGAGGCTG-3’
CATHEPSIN L	Forward	5’-ACCAAGTGGAAGGCGATG-3’
	Reverse	5’-TTCCCTTCCCTGTATTCCTG-3’
NeuN	Forward	5’-GGGGCTCCAAGGGTTTTG-3’
	Reverse	5’-GCCGACCACTGGATTTAGCT-3’
MAP2	Forward	5’-CCACCTGAGATTAAGGATCA-3’
	Reverse	5’-GGCTTACTTTGCTTCTCTGA-3’
NEF-L	Forward	5’-AGACCCTGGAAATCGAAGCA-3’
	Reverse	5’-TCACGTTGAGGAGGTCTTGG-3’
SYP	Forward	5’-CAAGGGGCTGTCAGATGTGA-3’
	Reverse	5’-CCTGTCTCCTTAAACACGAACC-3’
GAD67	Forward	5’-TCAAGTAAAGATGGTGATGGGATA-3’
	Reverse	5’-GCCATGATGCTGTACATGTTG-3’
vGLUT2	Forward	5’-GACCTACCCAGCATGTCATG-3’
	Reverse	5’-ACCAGACCATTCCAAAGCTTC-3’
GAPDH	Forward	5’-TCCTCTGACTTCAACAGCGA-3’
	Reverse	5’-GGGTCTTACTCCTTGGAGGC-3’

TABLE S2: LIST OF ANTIBODIES

Table S2. List of antibodies.

Antibody	Host	Company	Cat. No	WB	IF
Cystatin B	Mouse Monoclonal	R&D System	MAB1408	1:250	-
Cathepsin B	Mouse Monoclonal	Abcam	ab58802	1:500	1:200
Cathepsin D	Mouse Monoclonal	Abcam	ab6313	1:1500	1:200
Cathepsin L	Mouse Monoclonal	Santa Cruz Biotechnology	sc-32320	1:50	1:100
Parp1	Rabbit Monoclonal	Cell Signaling Technology	9532	1:1000	-
Gapdh	Rabbit Polyclonal	Bioss Antibodies	bs-10900R	1:1000	-
Cystatin B	Rabbit Polyclonal	Atlas Antibodies	HPA017380	-	1:350
Neurofilament	Rabbit Polyclonal	Abcam	ab8135	-	1:1000
Map2	Mouse Monoclonal	Thermofisher Scientific	MA5-12826	-	1:1000
Anti-Mouse IgG AlexaFluor® 488	Goat Polyclonal	Life Technologies	A-11001	-	1:500
Anti-Rabbit IgG AlexaFluor® 594	Goat Polyclonal	Life Technologies	A-11002	-	1:500
